# Fibroblast Lineage Switching as the Developmental Origin of Scarring and Target for Regenerative Healing

**DOI:** 10.3390/biology15050409

**Published:** 2026-03-02

**Authors:** Argyri Niti, Kokkona Kouzi-Koliakou, Anna Michopoulou

**Affiliations:** 1 Biohellenika Biotechnology Company, 57001 Thessaloniki, Greece; argyrw.niti@gmail.com (A.N.); kouzi@biohellenika.gr (K.K.-K.); 2 Department of Biochemistry, School of Medicine, Aristotle University of Thessaloniki, 54124 Thessaloniki, Greece

**Keywords:** skin, wound healing, scar, regeneration, fibroblast

## Abstract

Responses to cutaneous injury differ fundamentally across developmental stages in several mammal species. During early human gestation, when the fetus is less than 24 weeks old, wounds are capable of restoring normal tissue architecture without forming fibrotic scars. In contrast, postnatal and adult injuries typically resolve through the process of fibrosis. This divergence reflects coordinated differences in epidermal and dermal compartments, inflammatory signaling, extracellular matrix (ECM) composition, mechanical cues, and gene regulation. Recent studies have demonstrated that dermal fibroblasts are no longer considered a uniform population but instead arise from distinct developmental lineages with stable functional identities. Engrailed-1-negative fibroblasts (ENFs) predominate in early fetal skin in mice and support regenerative repair, while Engrailed-1-positive fibroblasts (EPFs) emerge later in development and are the principal contributors to fibrotic matrix deposition following injury. The developmental shift between these fibroblast populations coincides with the loss of scar-free healing capacity. This review examines the current understanding of fibroblast lineage specification, with particular emphasis on the roles of mechanotransduction, extracellular matrix cues, and epigenetic regulation. Elucidating how these lineage-encoded programs are established and maintained may enable strategies to reprogram adult fibroblasts toward a fetal-like regenerative state and thereby promote scar-free tissue repair.

## Simple Summary

This review discusses the most recent advances in the mechanisms that drive the transition from regenerative healing to scarring that is observed during development. Wounds in early-gestation embryos of less than 24 weeks of age heal through regenerative mechanisms that restore normal tissue architecture and form new appendages, such as hair follicles, without scarring. This divergence reflects coordinated differences in epidermal and dermal compartments, inflammatory signaling, extracellular matrix (ECM) composition, mechanical cues, and gene regulation. This review focuses on the changes observed in the behavior of different lineages of fibroblasts during development as central regulators of scar tissue formation. Elucidating how these lineage-encoded programs are established and maintained may enable strategies to reprogram adult fibroblasts toward a fetal-like regenerative state and thereby promote scar-free tissue repair.

## 1. Introduction

The skin functions as a multifunctional organ that provides mechanical protection, regulates fluid balance, and mediates immune surveillance. Structurally, it consists of a stratified epidermis anchored to an underlying dermis rich in ECM and mesenchymal cells. Following injury, the skin initiates a coordinated repair response aimed at restoring barrier integrity and preventing infection or fluid loss [1].

Cutaneous wound healing is traditionally described as a sequence of partially overlapping phases, including inflammation, proliferation characterized by tissue formation, and remodeling [2]. Animal studies in mammalian embryos have demonstrated that injuries sustained during early fetal development (<E16 in mice) typically heal through the complete restoration of native tissue architecture, including the regeneration of hair follicles and glands, without visible scar formation [3,4,5,6,7]. Most mammals, including mice, rabbits, lambs, and sheep, lose this regenerative capacity after mid-gestation, and healing increasingly resembles the fibrotic response observed in adults [5,8,9,10]. Human fetal skin demonstrates scarless healing before approximately 24 weeks of gestation, as demonstrated in vivo [7] and in in vitro human models [6].

Skin repair is the result of dynamic and interactive processes that involve soluble factors, ECM components, blood elements, and cells. Re-epithelialization is a critical step in the wound-healing process since defects in the formation of new epidermis lead to chronic non-healing wounds [11]. In human adults, wounds contract because fibroblasts pull on the ECM [12]. As the inflammatory phase concludes, fibroblasts enter the wound to initiate granulation tissue formation, which, in adults, is the precursor to a mature scar. Due to exposure to transforming growth factor-β (TGF-β) and the ECM of adult wounds, fibroblasts take on a contractile phenotype named myofibroblasts. This is a critical step in the adult wound-healing process, which contributes to wound closure via contraction [13]. Despite initial thoughts that embryonic fibroblasts were incapable of turning into myofibroblasts, experimental data concluded that, although ephemeral, a transition of embryonic fibroblasts to myofibroblasts was possible after exposure to large amounts of TGF-β1 in vitro [14,15]. Nevertheless, induced embryonic myofibroblasts produced less collagen, whereas the transplantation of embryonic fibroblasts to adult wounds resulted in healing with a reticular collagen pattern, indistinguishable from that of healthy skin [16]. In fact, mammalian embryos use rapid re-epithelialization in the absence of extensive inflammation and granulation tissue formation. This is a scarless process driven by the rapid formation of a contractile actin–myosin “purse-string” cable at the wound margins that pulls cells together to seal the gap [17,18].

Hence, a central challenge in regenerative medicine is elucidating the mechanisms underlying fetal skin regeneration following injury, as opposed to adult wound scarring, and reactivating these regenerative mechanisms for therapeutic purposes. Historically, research has focused on studying and analyzing the differences in inflammatory responses, ECM composition, growth factor profiles, and levels of mechanical tension between fetal and adult skin in different mammalian species [19,20,21,22]. Although each of the mentioned factors contributes to the overall outcome, they do not completely explain why the fetal environment allows regeneration, nor how these permissive conditions are lost during development.

An initial hypothesis was that fetal wounds heal without scarring because they develop in a sterile, protected uterine environment with minimal microbial exposure. Fetal sheep wounds were created in utero and compared across gestational ages. Despite being in the same sterile intrauterine environment, early-gestation wounds healed without scars, whereas later-gestation wounds formed scars [4,5]. Moreover, several studies provided evidence that the reduced inflammation observed in early fetuses cannot be solely responsible for scarless healing [23,24]. Similarly, lower mechanical tension may influence fibrosis severity but cannot fully account for regenerative healing. Therefore, environmental factors were excluded as the cause of scarless repair, and it was suggested that scarless healing is most likely an intrinsic property of early embryonic skin [25]. However, intrinsic programming likely emerges within a coordinated developmental context characterized by immune immaturity, matrix compliance, and distinct growth factor balance. Thus, environmental factors may not be independent causes of but rather contributors to the establishment of a developmentally encoded fibroblast phenotype.

This suggestion is in line with the relatively recent evidence that distinct fibroblast lineages exist in all mammals’ skin, representing unique cell types, and their switch from one type to the other during development induces the transition from regeneration to scarring [26,27,28]. Since the early 1990s, the influence of the environment has been assessed as the principal factor that may influence fibroblast behavior and the initiation of scarring in adult healing. Recent lineage-tracing studies have introduced a different concept. Data from mice strongly demonstrate that fibroblast identity—and more specifically, the developmental switch from ENFs to EPFs—is most likely the primary determinant of whether a wound heals through regeneration or fibrosis. ENFs, which dominate early fetal skin, are intrinsically pro-regenerative. EPFs, which emerge later in development and dominate postnatally, are intrinsically profibrotic and responsible for scar ECM deposition. This lineage switch appears to coincide with the loss of the scarless healing property, suggesting that fibroblast ontogeny may be a compelling mechanistic driver of the regeneration-to-fibrosis transition [27,28,29,30].

Lineage-driven fibrosis is well-documented in animal (mice) models and is mechanistically linked to conserved signaling pathways. Previous studies have revealed different subpopulations of functionally distinct fibroblast subpopulations in developing mice [26,27,31]. Multiple fibroblast subpopulations were also identified in adult mouse skin, presenting an adaptation of their abundance to the different phases of the hair growth cycle [32]. Although direct developmental lineage tracing is not feasible in humans, data strongly support fibroblast heterogeneity in humans [33,34,35,36,37,38]. Chang et al. previously showed that human fibroblasts display distinct, anatomically determined gene expression profiles depending on their body site of origin. These site-specific transcriptional programs persist during in vitro culture, demonstrating intrinsic positional memory that likely contributes to regional differences in tissue repair and disease susceptibility [39]. In a recent study, Morioka et al. identified eight distinct fibroblast subpopulations in the early embryonic human dermis (8 to 17 weeks) by analyzing single-cell RNA sequencing data and using single-molecule FISH to map their spatial distribution and interactions. The study concluded that fetal clusters did not directly map onto adult clusters, but there was a clear relationship between at least some of them [35]. Collectively, these data suggest that, similarly to mice, some human fetal fibroblast subpopulations could be used to promote scar-free healing in the future. Recent data also provide evidence that the fibroblast lineage specification observed in mice is likely mechanistically relevant to human fibrosis. In one study, Györfi et al. in 2021 demonstrated that Engrailed-1 coordinates cytoskeletal reorganization to induce myofibroblast differentiation and thus provides a bridge between murine developmental models and fibrotic signaling in humans [40]. Understanding the signals behind fibroblast lineage specification may therefore unlock the ability to reprogram human adult fibroblasts into a fetal-like state. Such reprogramming would challenge the presumed irreversibility of adult fibrosis and enable therapeutic strategies that promote true tissue regeneration.

The cumulative body of evidence reviewed here supports that fibrosis is most likely not an inevitable outcome of tissue repair but rather the consequence of the developmentally encoded fibroblast identity interacting with biochemical and biomechanical cues. Genetic lineage-tracing studies demonstrate that fibrotic extracellular matrix deposition is lineage-dependent and, importantly, not strictly required for wound closure.

This review summarizes current knowledge on the developmental origin of fibroblast heterogeneity, the molecular and mechanical factors driving lineage switching, and emerging strategies for recapitulating fetal-like healing in adults.

## 2. Overview of Adult Wound Healing vs. Fetal Regenerative Healing

### 2.1. Fetal vs. Adult Wound Healing: A Comparative Overview

Scarless healing in humans is observed before approximately 24 weeks of gestation, after which injuries begin to heal with progressively more adult-like fibrosis [6,7,21]. Withholding the healing of fetal wounds during this early window results in the following typical characteristics: 1. rapid coverage with new epithelium without the formation of granulation tissue, which is correlated with scar tissue formation postnatally [41]; 2. restoration of skin appendages, including hair follicles [42]; and 3. regeneration of the complete collagen architecture rather than deposition of aligned bundles [3,43]. Consequently, adult cutaneous wound healing results in incomplete regeneration of the original tissue, excessive production of an unorganized collagen meshwork that forms a scar, loss of appendages, and a flattened epidermis. In fact, the newly formed tissue has a lower tensile strength of less than 70–80% [42,44].

The progress of biological events during the wound-healing process, i.e., hemostasis, inflammation, proliferation, and remodeling, does not depend on age. However, the quality, magnitude, and timing of these processes are profoundly different [20,25].

Hemostasis, by definition, means to stop the hemorrhage. When a trauma occurs, this is achieved via the formation of a fibrin clot, which is created by the platelets leaking from the disrupted vasculature. At the same time, the platelets degranulate and release cytokines and growth factors that attract other types of inflammatory cells to the wound site. The latter is what induces the next phase, namely, the inflammatory phase. The first inflammatory cells to be recruited are neutrophils and monocytes, followed by macrophages [25]. These inflammatory cells clear the wound bed from microorganisms and tissue debris but also release chemoattractant signals that act on adaptive immune cells (such as mast cells, dendritic cells, and T-lymphocytes [45]), fibroblasts, and endothelial cells to form granulation tissue [13,25]. As the inflammatory phase progresses, fibroblasts from the reticular dermis [46] adhere to fibronectin deposited within the fibrin clot and reach the wound. There, they start producing and depositing granulation tissue [47] rich in collagens and other ECM components, such as hyaluronic acid (HA), fibronectin (FN), and tenascin C [48]. Subsequently, in response to TGF-β1 signaling originating from the immune cells and likely Wnt/β-catenin signaling [46,49], as well as the ECM of the wound bed, fibroblasts from the reticular dermis are activated and turn into contractile myofibroblasts [14,46,50]. In postnatal wound healing, the transition of fibroblasts to myofibroblasts is critical for wound closure via contraction of the wound edges achieved through interaction with the ECM [49]. The formation of granulation tissue is a hallmark of the initiation of the proliferative phase that aims to replace the destroyed tissue. One essential event in this phase is the activation of keratinocytes, which simultaneously migrate over the granulation tissue and proliferate to compensate for the loss in cell number, leading to the formation of new epithelium, the so-called re-epithelialization phase [25,51]. The wound-healing process concludes with the remodeling phase, which occurs 2 to 3 weeks post-injury and results in the formation of scar tissue. This phase is characterized by a reduction in the cell component of the wound milieu via apoptosis, rearrangement of collagen fibers, regression of neovascularization, and the deposition of components of the ECM [45,52]. The scar tissue presents a different orientation of collagen, a disorganized elastic fiber network, more dermatan sulfate, and less hyaluronic acid (HA) as compared to the normal skin, making it less extensible [52].

As for early-gestation fetal wounds, a markedly different trajectory is followed. Scar formation is considered a solution to the age-related, delayed re-epithelialization process. However, fetal wound healing is characterized by the remarkably rapid regeneration of the injured epithelium [25]. Overall, inflammation is limited in both scale and persistence, granulation tissue formation is minimal or absent, and tissue replacement occurs without the prolonged activation of fibrogenic pathways. Evidence suggests that fetal keratinocytes present different properties and use a different mechanism to migrate to and resurface the disrupted area. Not only do they proliferate faster, but they also form a cable that runs from cell to cell and contracts the keratinocytes together to close the wound within hours of wounding [25]. As a result, fetal wounds resolve with restoration of normal dermal and epidermal organization, an outcome that has been observed across multiple mammalian species [21]. Early assumptions attributed this regenerative outcome to environmental factors such as reduced immune activation or the sterile intrauterine milieu. However, experimental evidence demonstrating scarless healing in fetal skin transplanted into adult environments has challenged this view, suggesting that regenerative capacity is governed primarily by the intrinsic properties of the tissue itself [53]. Interestingly, strong evidence suggests that scarless wound healing in the oral mucosa is also derived from intrinsic differences in the tissue rather than from the environment [42].

As described in detail in the following sections, fetal wound healing reflects a coordinated biological state in which inflammation is muted, the ECM is soft and hydrated, cytokine signals favor regeneration, and fibroblasts exist in a plastic, pro-regenerative state. In adult skin, these parameters shift toward inflammation, mechanical stiffness, and profibrotic signaling, creating an environment that supports scarring. Therefore, the capacity of early-gestation human embryos’ skin to heal without scarring reflects a fundamentally different biological program to the fibrotic wound healing observed in adults [21,25,54]. The contrasting outcomes of fetal and adult repair, therefore, reflect not merely quantitative differences in wound-healing responses but the engagement of fundamentally different tissue programs.

### 2.2. Inflammatory Response

One of the most prominent biological distinctions between regenerative fetal and fibrotic postnatal wound healing is the nature of the inflammatory response [41,42,52].

The inflammatory response is a prerequisite for successful wound healing in adults [2], and it initiates within a few minutes to hours upon injury. Postnatal healing initiates with a robust inflammatory response with high neutrophil and macrophage infiltration and the release of proinflammatory cytokines and growth factors with pleiotropic roles in activating and/or regulating cellular responses. Initially, disruption of the vasculature drives platelet aggregation from the circulation into the wound site and formation of the clot [52]. The latter is accompanied by the degranulation of platelets and the release of platelet-derived growth factor (PDGF), TGFβ1, tumor necrosis factor α (TNFα), and interleukin-1 (IL-1), which activate resident cells and recruit macrophages to the wound site [41,42]. The proinflammatory cytokines IL-6 and -8 are also produced during the inflammatory phase. These signals further induce activation of the migratory phenotype in keratinocytes at the wound edges and fibroblast recruitment and transformation to myofibroblasts, which, in turn, collectively release more cytokines and growth factors acting in an autocrine or paracrine manner [2,20,52]. Vascular endothelial growth factor (VEGF) is also upregulated in adult wounds, mediating wound angiogenesis [42]. Therefore, inflammatory cells may play a central role during the inflammatory stage; nevertheless, non-inflammatory cells and the ECM that they secrete also contribute.

The fetal wound-healing process is characterized by a minimal inflammatory response. The main differences are recapitulated as follows: To begin with, platelets exhibit variations depending on the stage of gestation. There is evidence suggesting that platelet aggregation and degranulation are decreased in early-gestation embryos. As a result, lower levels of TGFβ and PDGF are released at the wound site. Some in vitro studies have shown that platelet-rich plasma (PRP) produced by platelets possibly induces dermal fibroblast differentiation to myofibroblasts [55]. However, contradictory results from different studies do not allow us to draw definitive conclusions. In turn, less TGβ1 has been correlated with reduced neutrophil recruitment [56]. Moreover, levels of the proinflammatory IL-6 [24,57] and IL-8 [23] are characteristically lower in scarless healing, contributing to the lower acute inflammatory infiltrate at the wound bed. Overall, fewer macrophages, neutrophils, dendritic cells, mast cells (degranulating less effectively), T cells, and Langerhans cells are detected in fetal tissue [21,58,59,60], while macrophages are mostly represented by the M2 (anti-inflammatory) phenotype [61,62,63]. In early-gestation fetuses, macrophages are not recruited to fetal wound sites due to lower expression levels of TGFβ1, which contributes to the conversion of circulating monocytes to macrophages [42]. Instead, in scarless wounds, there is another version of TGFβ, i.e., TGFβ3, whose expression is elevated, and its presence has been linked to hypoxia [64] and leads to an inhibitory signal for terminal differentiation [65]. It is likely that the presence of TGFβ3 keeps the tissue in an immature state that impacts the release of proinflammatory cytokines and eventually favors healing with minimal scar formation [66]. Another cytokine detected in scar-forming mice that induced scar formation in scarless embryo wounds in an experimental setting is IL-33 [56]. On the contrary, the anti-inflammatory cytokine IL-10 is highly expressed in fetal wounds. Finally, fibroblasts that actively participate in immune regulation present age-related differences in the expression of major histocompatibility complex (MHC) classes I and II in humans [52].

The relative absence of inflammation in fetal skin is thought to protect fibroblasts from adopting a profibrotic identity and to maintain a pro-regenerative milieu.

### 2.3. Extracellular Matrix Composition

**Figure 1:**
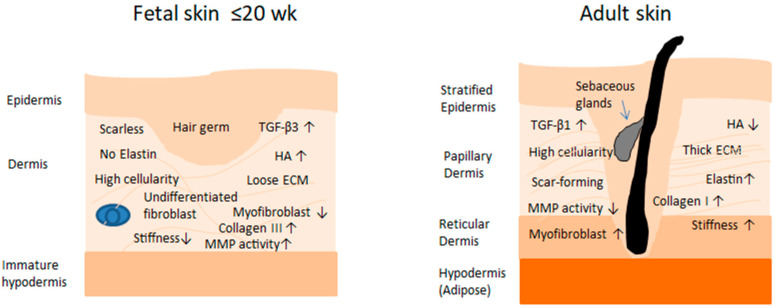
Comparison of embryonic (≤20 weeks) and adult human skin architecture and extracellular matrix (ECM) composition. ↑ Increase, ↓ Decrease.

Marked differences in the composition and structural organization of the ECM characterize fetal versus adult wound healing (Figure 1). These variations are critical because they fundamentally alter the biochemical milieu and biomechanical properties of the matrix. In doing so, they shape the quality and identity of the newly formed tissue, influence the behavior and phenotypic state of the cells participating in repair, and modulate the complex network of mechanical and molecular cues that orchestrate the wound-healing response [22].

Collagen is a key structural component of the skin, both in the fetal and later stages, with type one collagen predominating in all phases. Its mechanical robustness is derived from a three-stranded helical arrangement of polypeptide chains, whose crosslinking and stabilization are mediated by the activity of lysyl oxidase [47]. However, in fetal skin, there is a predominance of collagen III και V over collagen I, which decreases over time. Correspondingly, fetal fibroblasts show an increased ratio of collagen III and IV to collagen I, compared to neonatal and adult fibroblasts (rats, sheep, and humans) [21,39,67,68,69]. Additionally, increased expression was observed in collagens IV and XIV in fetal versus neonatal and adult human fibroblasts [39,70]. The specific collagen profile within the ECM plays a central role in directing fibrillogenesis, thereby determining both the diameter of individual fibrils and the organization of collagen bundles. Collagen type I promotes the formation of relatively thick fibrils and is associated with a slower turnover compared with type III. Type V is required for the proper assembly of collagen I and III fibrils, while collagens V and XIV function as regulatory elements that constrain the ultimate diameter of the collagen fibers [71,72]. This explains the fact that, in fetuses, collagen deposition occurs in thin, reticular, and more organized bundles, while in adults, it occurs in denser and parallel bundles [73]. By extension, it is understood that, while the crosslinking of type I collagen is critical for conferring tensile strength during adult wound repair, the resulting matrix stiffness can restrict the mobility of key cellular mediators. In fetal tissues, this increased rigidity may hinder the swift cellular dynamics required for accelerated regenerative responses [74]. Moreover, data support that the more rapid wound healing in fetuses as compared to adults is achieved through the earlier secretion of higher amounts of total collagen (collagen types I, III, IV, V, and VI) [22].

Other major components of the ECM are glycosaminoglycans (GAGs), particularly hyaluronic acid (HA) and chondroitin sulfate (CS) [21,75]. HA is a negatively charged, unsulfated glycosaminoglycan found in a soluble form or complexed with proteoglycans, which increases during rapid cellular migration and angiogenesis. The overall negative charge of hyaluronic acid enables it to attract and transiently restrain water molecules, a property that contributes to resistance against mechanical deformation while simultaneously supporting efficient cell migration through the matrix and stimulating collagen synthesis by fibroblasts [47]. The HA content in scarred fetal wounds increases more rapidly than that in scarred adult wounds, while fetal fibroblasts also present higher amounts of HA receptors, which allows them to maintain the amount of HA produced for longer and promotes their migration [76]. Furthermore, the increased expression of HA in embryos reduces the uptake of inflammatory cytokines such as IL-1 and TNF-alpha [77].

Proteoglycan-associated ECM regulators such as decorin and fibromodulin, along with enzymes including lysyl oxidase and the matrix metalloproteinases (MMPs), collectively participate in orchestrating the processes of collagen production, post-translational maturation, and controlled degradation [78]. Decreased expression of decorin and increased expression of fibromodulin have been observed after injury in early gestation compared to in later developmental stages [79]. Reduced decorin has been shown to cause reduced tensile strength [80] and regulate collagen fiber formation, while fibromodulin has been associated with anti-inflammatory function and increased cellular migration [81]. In general, embryonic fibroblasts exhibit elevated levels of enzymes involved in collagen crosslinking—including lysyl hydroxylase (LH), prolyl hydroxylase (PH), low lysyl oxidase (LOX) [82], and transglutaminase 2 (TGM2) [70]—a pattern of expression that is accompanied by enhanced collagen production [22]. LOX is expressed at higher levels during adult tissue repair, and its upregulation has been associated with the development of fibrotic pathologies [83]. Nevertheless, findings from another investigation reported that fetal wounds exhibit higher LOX expression than neonatal wounds, indicating that additional studies are needed to clarify this discrepancy [84]. Furthermore, the expression of MMPs is necessary for the release and migration of cells from the ECM. A higher ratio of MMP to TIMP expression has been observed to be associated with scarless repair in rats [85].

Fetal wounds that heal without scarring exhibit a faster induction of ECM adhesion molecules and a distinct pattern of integrin expression on the cell surface. During early human embryogenesis, elastin is present at minimal levels because its synthesis peaks in the late embryonic and neonatal periods and subsequently declines, resulting in limited regenerative capacity in adult tissues [21,75]. Conversely, FN is more abundant in fetal skin, preceding and facilitating the deposition of tenascin, a matrix component that suppresses cell adhesion and emerges earlier in fetal repair [21,43]. Laminin expression remains stable across developmental stages in human fibroblasts [86]. Moreover, fetal fibroblasts display elevated levels of integrin α2 and reduced levels of α1 and α3 compared with adult cells, a profile associated with their diminished ability to contract collagen matrices [87].

It is important to recognize that scarless fetal healing is not uniform across all tissues. During early gestation—when the skin is capable of regenerating without fibrosis—organs such as the fetal stomach, intestine, and diaphragm nonetheless undergo scar formation following injury. This disparity implies that specific skin-resident cell populations play a crucial role in directing the localized wound-healing response [88,89]. Conversely, evidence indicates that the uterine (endometrial) environment alone does not determine, nor can it independently induce, a scarless pattern of repair [53,90].

The fetal ECM is compositionally distinct from the adult ECM and plays a major role in influencing fibroblast behavior. The ECM most likely does not merely reflect fibroblast activity but actively drives it: fetal ECM provides cues that support regenerative fibroblast phenotypes, while adult ECM reinforces profibrotic behavior. The question that arises here, though, is what and through which mechanism produces this ECM, given that one population of fibroblasts is favored over the other.

### 2.4. Biochemical Signaling Through the ECM

Beyond its structural role, the extracellular matrix functions as a dynamic biochemical signaling platform that instructs fibroblast fate and behavior. Fibroblasts actively sense ECM composition, organization, and stiffness through integrins, proteoglycan receptors (such as syndecans), and mechanosensitive ion channels, translating extracellular cues into intracellular signaling cascades that regulate proliferation, migration, differentiation, and fibrotic versus regenerative outcomes.

Members of the transforming growth factor family play a central role in the regulation of wound repair. TGF-β functions as a potent chemoattractant for fibroblasts, keratinocytes, and immune cells while also promoting collagen type I synthesis by fibroblasts [91]. In fetal wounds, TGF-β3 is expressed at markedly elevated levels; this factor, produced mainly by keratinocytes and fibroblasts, is closely associated with skin morphogenesis. By contrast, the expression of TGF-β1 and TGF-β2 remains minimal in fetal repair. Adult wounds display an opposing profile, being dominated by TGF-β1 and TGF-β2, which initially originates from platelet degranulation and is later supplied by infiltrating inflammatory cells, including monocytes and macrophages [92].

In this context, TGF-β acts as a regulator of MMP expression. TGF-β1 inhibits MMPs, mainly MMP1 [93]. MMPs mediate the proteolytic breakdown of ECM, and their activity within tissues is tightly controlled by TIMPs. The dynamic equilibrium between these enzymes and their inhibitors is a critical determinant of tissue remodeling, as it governs the turnover and persistence of the ECM. Embryonic wounds are characterized by an elevated MMP-to-TIMP ratio, resulting in enhanced degradation of ECM components. This enzymatic environment promotes dynamic matrix remodeling rather than excessive ECM deposition at the site of injury [94]. Similarly, adult wound environments are enriched in PDGF, a factor that is largely absent from embryonic wounds due to minimal platelet degranulation. In contrast, embryonic wounds exhibit elevated levels of endogenous fibroblast growth factors (FGFs) that are associated with skin morphogenetic processes [95]. VEGF, a potent mitogen for endothelial cells, is upregulated approximately two-fold in wounds that heal without scarring, whereas its expression remains unchanged in fetal wounds that undergo fibrotic repair. This enhanced angiogenic and permeability-promoting signal may contribute to the accelerated healing observed in scarless fetal wounds [96].

ILs constitute a class of cytokines that play key roles in directing inflammatory cell recruitment and activation, thereby contributing to the regulation of wound repair. Notably, early embryonic fibroblasts exhibit a markedly reduced expression of IL-6 and IL-8, both under basal conditions and following PDGF stimulation, when compared with adult fibroblasts [97]. In addition, fetal skin demonstrates higher levels of IL-10 expression than adult tissue [98].

The integrin–focal adhesion kinase (FAK), a key cytoplasmic tyrosine kinase, regulates cellular processes such as proliferation, survival, and signaling and so represents a key regulator of mechanotransduction in the skin [99]. During wound repair, FAK is activated in response to mechanical stimuli and subsequently modulates intracellular signaling through multiple downstream effectors, including the PI3K and MAPK pathways, which are closely linked to fibrotic outcomes [100]. Inhibition of the FAK signaling pathway suppresses the activity of the MAPK pathway, reduces glycolytic metabolism in trophoblast cells, and negatively affects the healing process [99]. FAK upregulation is mainly observed in mature tissues and is associated with increased scar formation [101]. The transcriptional co-activators YAP and TAZ, targets of the Hippo pathway, are activated by mechanical stimulation transmitted through FAK and RhoA/ROCK, leading to their translocation to the nucleus and activation of genes associated with fibroblast activation, such as connective tissue growth factor (CTGF) [102]. In skin fibroblasts, YAP is nuclear in proliferating cells and is mainly cytoplasmic in quiescent cells after birth, while in early fetal skin fibroblasts, it is mainly localized in the nucleus [103]. Accordingly, YAP/TAZ signaling is preferentially activated in adult skin as a consequence of elevated mechanical tension and increased extracellular matrix stiffness—conditions that are largely absent in fetal and neonatal tissues [104]. During early developmental stages, YAP/TAZ activity is stringently controlled and remains attenuated within mechanically compliant, low-stiffness microenvironments [105]. Moreover, Rho/ROCK signaling converges with the Hippo pathway through its role in mechanically regulating YAP and TAZ activity. ROCK-driven actomyosin contractility increases cytoskeletal tension, facilitating the nuclear translocation of YAP/TAZ and the subsequent activation of transcriptional programs linked to cell proliferation and lineage commitment. Compliant extracellular matrices are associated with reduced ROCK signaling and lower intracellular tension, conditions that favor maintenance of stem cell characteristics. In stiffer adult matrices, enhanced cellular contractility promotes YAP/TAZ activation, biasing stem cell fate toward epithelial or mesenchymal differentiation [106].

Wnt signaling plays a critical role in embryonic development and organogenesis. Wnt-responsive cells in dermal tissue include hair follicle bulge cells, basal interfollicular epidermal cells, and dermal fibroblasts [19]. In adult skin tissue, several Wnt proteins (Wnt 1, 3, 4, 5, and 10) are activated during normal wound healing, similar to cell proliferation signals for dermal fibroblasts and keratinocytes. In contrast, in fetal skin, Wnt4, Wnt5a, and Wnt11 are expressed in the dermis and play an important role in hair follicle morphogenesis [107].

### 2.5. Gene Expression and Transcriptional Programs

The transition from regenerative to fibrotic wound healing has been proposed to reflect, at least in part, a developmental compromise that compensates for the comparatively delayed re-epithelialization observed in adult skin relative to fetal skin. However, unveiling cause–effect relationships in this process remains challenging. Here, we provide an overview of differences in gene expression, transcriptional programs, and signaling cues that influence keratinocyte and fibroblast behavior in fetal versus adult skin. Keratinocytes are discussed because of their capacity to modulate fibrotic responses, whereas fibroblasts represent the principal extracellular matrix-producing cells and play a central role during the proliferative and remodeling phases of wound healing.

During fetal skin development, the epidermis transitions from a single-layered structure at early gestation into a stratified and keratinized epithelium by gestational weeks 22–24 [78]. At approximately 14 weeks of gestation, the fetal epidermis consists of a basal layer, one or two intermediate layers, and the periderm [21]. At this early stage, highly proliferative keratinocytes progressively commit to stratification, a process reflected by dynamic changes in keratin expression. Keratins K5 and K14 are selectively expressed in proliferating basal keratinocytes, and K1 and K10 are induced at weeks 9–10 and mark keratinocytes committed to terminal differentiation in the suprabasal layers [21]. Additional keratins such as K8, K17, and K19 are preferentially expressed in fetal keratinocytes and used as markers to distinguish them from adult keratinocytes in vitro [21,108].

Functionally, fetal keratinocytes display an enhanced proliferative capacity in vivo [21] and increased clonogenicity in vitro, characterized by longer telomeres and decreased expression of major histocompatibility complex (MHC) proteins [108]. Notably, fetal keratinocytes do not induce proliferation of naïve T cells, suggesting a mechanism of immune tolerance at the maternal–fetal interface during pregnancy. Consistent with this observation, fetal keratinocytes produce higher levels of antimicrobial peptides than postnatal keratinocytes, including β-defensins, S100 protein family members, and cathelicidin, indicating that innate antimicrobial defense represents a critical protective strategy in the embryo [109].

At the transcriptional and epigenetic levels, fetal keratinocytes exhibit distinct chromatin landscapes. Reduced levels of trimethylated histone H3k27 and an increased expression of histone demethylase JMJD3 have been reported in fetal compared with postnatal and adult keratinocytes [109,110,111]. In contrast, postnatal keratinocytes display an increased activation of Wnt signaling pathways [112]. Nuclear accumulation of β-catenin following Wnt-3α signaling promotes transcriptional activation via TCF/LEF enhancers and has been linked to profibrotic gene expression [113,114]. Additionally, members of the SRY-related high-mobility-group (HMG) box (SOX) family of transcription factors, particularly SOX-4 and SOX-11, regulate epidermal differentiation during embryogenesis through mechanisms that involve AP-1 transcription activity [115]. During adult wound healing, reactivation of embryonic gene programs regulating keratinocyte migration has been shown to involve these transcription factors [116].

Accumulating evidence suggests that many of the fundamental differences between fetal and adult skin reside within the dermal compartment, implicating fibroblasts as key effectors of scarless repair. Supporting this concept, keratinocytes cultured on fetal human dermal fibroblasts present an increased expression of cell cycle-associated genes, while adult fibroblasts promote keratinocyte differentiation, reinforcing the idea that age-dependent changes in the extracellular matrix critically influence wound-healing outcomes [117]. Fetal fibroblasts proliferate and migrate more rapidly than adult fibroblasts while simultaneously synthesizing collagen [22,75]. These functional differences are partially attributable to distinct ECM composition and deposition kinetics. Fetal fibroblasts secrete higher amounts of total collagen, including collagen types I, III, V, IV, and XIV, with collagen I deposited more rapidly and at a lower percentage, resulting in thinner collagen fibers that favor scarless healing [22].

Proteomic analyses further revealed differences in cytoskeletal regulation and protein turnover between fetal and adult fibroblasts. Fetal fibroblasts express significantly higher levels of tubulin alpha 1 and actin, whereas adult fibroblasts exhibit an increased expression of actin-regulating proteins such as fructose-bisphosphate aldolase A, cofilin-1, and profilin-1, which may impair ubiquitin-mediated protein degradation and delay protein turnover. Moreover, fetal fibroblasts demonstrate enhanced superoxide radical degradation and early activation of apoptosis-related signaling pathways following injury, suggesting that efficient detoxification of reactive oxygen species and timely removal of damaged cells contribute to scarless repair [113,118]. In contrast, adult fibroblasts overexpress proteins associated with proinflammatory signaling, granulation tissue formation, and cell adhesion, such as lactotransferrin, galectin-1, and calreticulin-1 [119]. More recently, fibrotic repair has been linked to fibroblast expression of the transcription factor EN-1, increased YAP activity, and suppression of Trps1, highlighting the contribution of mechanosensitive and lineage-associated transcriptional programs to scarring outcomes [114,120].

Together, these findings indicate that developmental stage-dependent differences in gene expression, mechanotransduction, and epigenetic regulation progressively restrict transcriptional plasticity in skin cells. Rather than acting as transient modulators of wound repair, these processes establish stable transcriptional states that shape how dermal cells respond to injury. Such transcriptional and epigenetic priming provides the foundation for the emergence of fibroblast populations with distinct functional identities, which are discussed in the following section.

The dermal layer consists of three compartments with different architecture and collagen deposition patterns, namely, the papillary dermis, the reticular dermis, and the hypodermis, each containing different fibroblast populations that arise from varying lineages. Increasing evidence suggests that the dermis comprises at least two functionally distinct lineages of fibroblasts with various morphological and functional properties, as well as distinct roles in the wound-healing process (reviewed in [91]). Evidence suggests that papillary fibroblasts are likely important contributors to the scarless healing process [121]. Neonatal papillary fibroblasts lose their ability to form hair follicle mesenchyme upon injury, and, in turn, they are adapted to acquire their rapid wound-healing properties and become specialized in the fibrous deposition matrix once major tissue developmental processes are complete [122]. This phenomenon can only be converted at the center of large wounds. It appears that genetic upregulation of developmental pathways, such as Wnt and Shh, in fibroblasts enhances their ability to form hair follicles in adult skin. Evidence suggests that histone modifications are responsible for the regulation of the developmental maturation of fibroblasts. For example, Kim et al. showed that the transcription factor Twist 2 drives the decrease in acetylation of histone H3K27, which subsequently inactivates Wnt signaling, ultimately leading to postnatal fibroblast differentiation [122]. Other histone modifications, such as methylation of H3K27, seem to participate in the regulation of fibroblast maturation. Methylation seems to control the expression of αSMA and the differentiation to myofibroblasts [121]. Another possibility is that fibroblasts, similarly to keratinocytes, may carry epigenetic “memory” modifications that dictate their behavior upon injury. Interestingly, at the same time, adult fibroblasts exhibit remarkable plasticity and can convert to other cell types [121], although they demonstrate strong positional and behavioral stability when they are not perturbed [122]. Elucidating the origins of the distinct fibroblast subpopulations and the mechanisms that are responsible for their great plasticity is therefore of major importance.

## 3. Fibroblast Subpopulations and Lineage-Dependent Regulation of Wound Healing

The dermal layer of the skin is structurally and functionally heterogeneous, comprising distinct compartments—including the papillary dermis, reticular dermis, and hypodermis—that differ in extracellular matrix (ECM) organization, mechanical properties, and cellular composition [96,97,98]. Fibroblasts residing within these compartments exhibit distinct transcriptional, epigenetic, and functional identities that reflect their developmental origins [93,98,99,100,101,102]. Rather than constituting a homogeneous population, dermal fibroblasts comprise multiple developmentally encoded lineages whose relative abundance and activity change across ontogeny and in response to injury [91]. Understanding these lineage relationships is therefore central to explaining the transition from regenerative to fibrotic wound healing during development.

### 3.1. Embryonic Origin and Identification Markers of Dermal Fibroblast Subpopulations

Fibroblast heterogeneity arises from both embryonic origin and anatomical localization [121,123]. In most body regions, fibroblasts derive from mesodermal progenitors—originating from the lateral plate mesoderm or dermomyotome—whereas craniofacial fibroblasts arise from neural crest-derived ectoderm [124]. During skin development, multipotent mesenchymal progenitors populate the dermis and progressively differentiate into discrete fibroblast subsets under the influence of positional cues and morphogenetic signaling pathways, including Wnt, BMP, FGF, and Shh [125,126]. This maturation process coincides with compartmentalization of the dermis into papillary and reticular layers and with changes in ECM composition, mechanical properties, and appendage-forming capacity [35,125,127].

Many previous studies revealed the presence of multiple functionally distinct subtypes of fibroblasts in developing mouse skin. A combination of markers for the identification of multipotent mesenchymal cells with the ability to differentiate into all types of dermal fibroblasts includes PDGF receptor A (PDGFRA), delta-like homology -1 (DLK-1), EN-1, and leucine-rich repeat protein (LRIG1) [26,27,128]. After establishing a nascent connective tissue, skin progenitors undergo progressive specification into three main distinct groups, while these three groups are further separated into ten subgroups that display different combinations of gene expression patterns [121,124]. Currently, papillary fibroblasts (CD26+/SCA1+) and reticular fibroblasts (DLK1+/SCA1−) are established populations with distinct expression patterns and properties [121].

Although papillary and reticular fibroblasts can now be clearly discriminated from one another, studies have revealed that there are transcriptionally overlapped fibroblasts across skin layers [124]. Papillary fibroblasts seem to share a common progenitor with the erector pili muscle of the hair follicle and the dermal papilla fibroblasts that participate in the formation of the hair follicle [26]. Moreover, papillary fibroblasts are most likely related to scarless healing. Papillary fibroblasts express fap, CD26, Lrig1, integrin Itga8, and Blimp1. Adipocytes, adipocyte progenitor cells, and reticular fibroblasts are derived from common fibroblast progenitors expressing Pdgfrα, twist-related protein-2 (Twist-2/Dermo-1), and EN-1 [121]. To sum up, while papillary fibroblasts are associated with fine fibrillar matrix deposition and the support of hair follicle morphogenesis, reticular fibroblasts are more strongly linked to dense ECM deposition and fibrotic repair. Importantly, the transcriptional overlap between fibroblasts across dermal layers suggests that lineage identity is not strictly defined by location alone but reflects shared developmental trajectories [124].

Recent single-cell analyses have confirmed fibroblast heterogeneity in human fetal and adult skin [35,36,123]. In the fetal dermis, multiple fibroblast subtypes with distinct metabolic and biosynthetic profiles coexist, including progenitor-like populations that dominate early gestation and more differentiated fibroblasts resembling adult papillary and reticular populations [35]. These findings indicate that lineage diversification precedes birth and that fibroblast identities associated with fibrotic repair are established during development rather than arising solely in response to injury.

**Figure 2:**
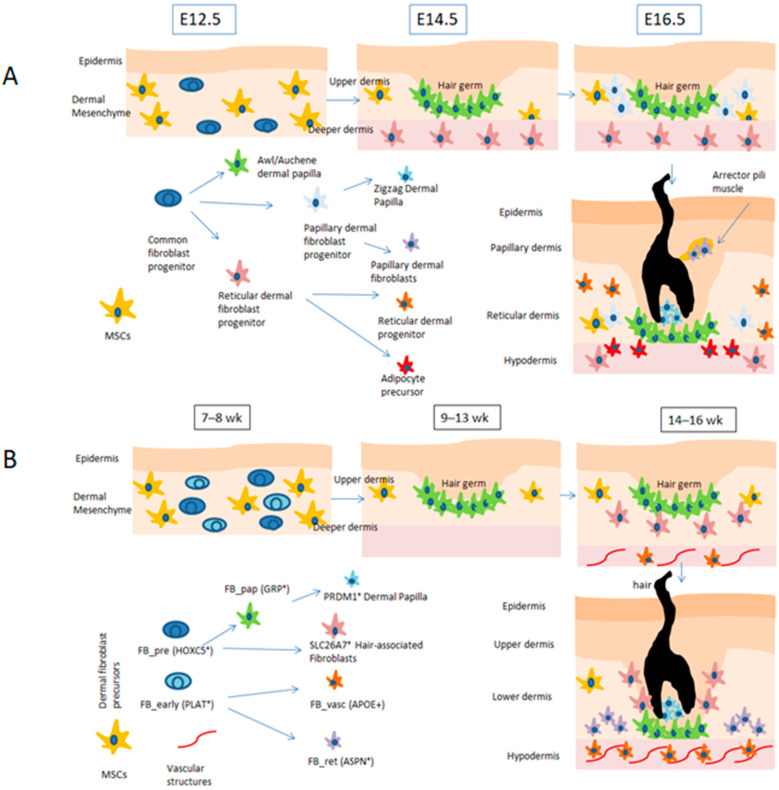
Comparative developmental trajectories of dermal fibroblast lineages in mouse (**A**) and human (**B**) skin. In mouse embryonic skin (E12.5–E16.5), a common fibroblast progenitor gives rise to papillary and reticular lineages that generate dermal papilla, papillary fibroblasts, reticular fibroblasts, adipocyte precursors, and arrector pili-associated cells during hair follicle morphogenesis. In human fetal skin (7–16 weeks after conception), HOXC5^+^ precursor (FB_pre) and PLAT^+^ early (FB_early) fibroblasts segregate into papillary (GRP^+^) and reticular (ASPN^+^) lineages, producing PRDM1^+^ dermal papilla, SLC26A7^+^ hair-associated fibroblasts, and APOE^+^ vascular-associated fibroblasts, as inferred from single-cell transcriptomic analysis. Schematic representation inspired by Driskell et al., 2013 [26], and Morioka et al., 2025 [35].

Fibroblast heterogeneity has also been recently confirmed in adult human skin [36,123,129] (Figure 2). In a study by Morioka et al., it was shown that at least eight subpopulations of fibroblasts reside within the fetal dermis with specific locations that correlate with their functions in developing skin. Two subpopulations designated as precursor (HOX5+) and early (PLAT+) fibroblasts were found as the predominant cell types at 7–8 weeks of gestation and exhibited highly active protein biosynthesis. Another subtype with high metabolic activity was related to the growth and development of HFs during embryogenesis. In contrast, the rest of the identified subtypes included vasculature-related fibroblasts, papillary and reticular fibroblasts with localization and gene expression similar to those in adult skin, and a distinct cluster of proliferative fibroblasts only identified in the fetal dermis [35].

### 3.2. Fibroblast Subpopulations Contribute Differently to Wound Healing

Lineage-tracing and functional studies in mice have demonstrated that fibroblast subpopulations contribute differentially to wound-healing outcomes [27,29]. Fibroblasts located in the papillary dermis have been associated with regenerative functions, including fine fibrillar matrix deposition and support of hair follicle neogenesis. In contrast, fibroblasts derived from deeper dermal compartments are more strongly linked to fibrotic matrix production and scar formation, as reviewed in [130]. These lineage-dependent behaviors are progressively established during development and reinforced after birth by epigenetic mechanisms that limit transcriptional plasticity. Recently, two pairs of embryonic fibroblast lineages have been identified with a significant role in scar formation in dorsal and ventral wounds. The first pair includes EPFs and ENFs. EPFs have been associated with scar formation in the dorsal wound. The second pair includes pair-related homeobox 1 (Prrx1)-positive (PPFs) and -negative fibroblasts (PNFs), with PPFs being responsible for scar formation on the ventral dermis of the mouse [27].

**Figure 3:**
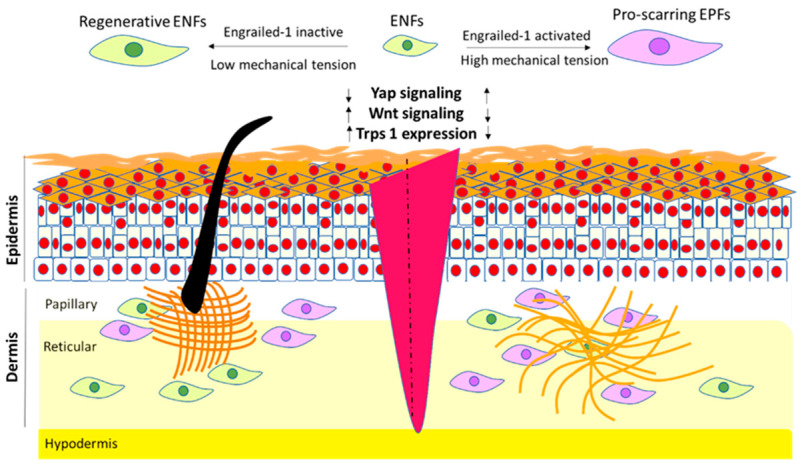
Activation of Engrailed-1 in response to mechanical tension in adult wounds. Mechanical tension applied to wounds leads to suppression of Trps 1 and induces transcriptional programs related to scarring outcomes. Wnt activation in the dermis is related to regeneration and hair follicle morphogenesis. Green fibroblasts: regenerative/ENF-like/papillary-associated; purple fibroblasts: fibrotic/EPF-like/reticular-associated. ↑ Increase, ↓ Decrease.

Functional lineage-tracing studies have provided direct evidence that fibroblast subpopulations contribute differentially to wound-healing outcomes (Figure 3). In the mouse skin, Engrailed-1 lineage-negative fibroblasts (ENFs) predominate in the early embryonic dermis and exhibit transcriptional programs associated with tissue plasticity, ECM remodeling, and regenerative competence. ENFs contribute to restoration of normal dermal architecture and appendage formation following injury. In contrast, Engrailed-1 lineage-positive fibroblasts (EPFs), which arise later in development and become dominant postnatally, display strong profibrotic behavior, robust ECM deposition, and a high propensity for myofibroblast differentiation, making them principal contributors to scar formation [27,28,120].

Lineage tracing reveals that ENFs and EPFs differ not only in developmental timing but also in spatial distribution, proliferative capacity, and sensitivity to mechanotransduction. In Table 1, the most well-established differences between ENF and EPF properties are presented. Notably, postnatal mechanical cues can drive ENF-to-EPF conversion through YAP/TAZ-dependent signaling pathways, indicating that fibroblast lineage identity is stabilized—but not irreversibly fixed—by mechanical and epigenetic inputs [40,131]. Complementary studies have identified analogous lineage-dependent fibroblast populations in ventral skin, including PRRX1-positive fibroblasts that similarly contribute to fibrotic repair [27].

## 4. Conclusions

Accumulating evidence suggests that the divergence between regenerative and fibrotic skin repair reflects developmentally encoded differences in fibroblast lineage identity that are likely enhanced by ECM composition and mechanical stimuli. Fetal wound healing is characterized by a compliant, collagen type III-rich, growth factor-retentive matrix that supports fibroblast plasticity and regenerative capacity, whereas adult repair occurs within a stiffer, collagen type I-dominant environment that promotes profibrotic fibroblast programs in response to mechanotransduction signaling [68,132,133].

Novel therapeutic strategies aim not only to suppress fibrosis but also to actively reprogram adult fibroblasts toward a regenerative, fetal-like state. These approaches target mechanotransductive signaling, extracellular matrix (ECM) composition, and epigenetic stabilization of fibroblast identity, reflecting the integrated nature of fibrotic commitment.

Mechanical signaling is central to adult scar formation. Increased matrix stiffness enhances integrin engagement and focal adhesion assembly, activating focal adhesion kinase (FAK), Rho/ROCK pathways, and downstream YAP/TAZ transcriptional programs that stabilize myofibroblast differentiation and collagen deposition [134,135]. Inhibition of FAK has been shown to significantly reduce scar formation in vivo by interrupting force-dependent profibrotic signaling pathways [136]. Similarly, inhibition of YAP/TAZ activity decreases α-smooth muscle actin (α-SMA) expression and extracellular matrix production [137]. Hence, treatment with verteporfin is an effective therapy for preventing fibrosis [131]. Since TGF-β-induced myofibroblast differentiation is enhanced in stiff matrices [14], targeting TGFβ is a rational strategy to recalibrate fibroblast activation. The TGFβ3 avotermin has previously been shown to reduce scarring in a phase I/II double-blinded, randomized clinical trial. However, it was finally stopped, as it was characterized as insufficient [138,139]. Application of angiogenin to target the TGFβ1/SMAD 2/3 signaling pathway was shown to attenuate scarring in vitro, although its safety is questionable [140].

Emerging biomaterial strategies, including heparin-functionalized collagen I/III scaffolds and engineered matrices designed to recapitulate embryonic ECM properties, demonstrate that it is possible to partially reconstitute key biochemical and mechanical cues of the regenerative niche [141,142]. By stabilizing and spatially presenting growth factors, modulating matrix composition, and tuning mechanical stiffness, such platforms can bias fibroblast behavior toward fetal-like, pro-regenerative states. Biomaterial-based approaches seek to recreate the compliant ECM environment characteristic of fetal skin. Matrix elasticity has been shown to direct lineage specification [143], and reduced collagen crosslinking diminishes fibroblast-generated tension and YAP/TAZ activation [137]. Verteporfin, which is a potent inhibitor of YAP/TAZ signaling, presents dose-dependent toxicity when it is administered systemically. Wang et al. developed bioadhesive nanoparticles for verteporfin encapsulation that enables topical delivery and sustained release [144]. Most recently, smart biomaterials acting at several levels and stages of wound healing have been developed. As an example, Zhang et al. produced a core–shell-structured microneedle array patch with anti-microbial properties, a core component neutralizing variable inflammatory factors, and simultaneous release of verteporfin [145].

Reprograming adult fibroblasts toward a fetal-like phenotype is another strategy. It has already been shown that inhibition of CD26 expressed by the majority of EPFs and fibroblast subpopulations involved in scarring, using MK0626, accelerated wound closure and decreased scar formation in vitro and in vivo [146]. Epigenetic stabilization constitutes a critical mechanism reinforcing fibroblast activation during adult fibrotic repair. Myofibroblast differentiation is associated with chromatin accessibility at profibrotic loci and altered histone acetylation dynamics. In human dermal fibroblasts, histone deacetylase (HDAC) inhibition suppresses TGF-β-induced collagen type I and α-SMA upregulation, demonstrating that acetylation status directly regulates fibrogenic transcriptional programs [147]. In systemic sclerosis, a prototypical fibrotic skin disorder, aberrant histone modification patterns, and increased HDAC expression contribute to sustained fibroblast activation [148], further supporting the role of epigenetic reinforcement in cutaneous fibrosis.

Despite encouraging preclinical evidence, major challenges remain. The human dermis contains heterogeneous fibroblast subpopulations with positional memory and lineage-specific behavior [35,36,130]. Selective targeting of fibrogenic subsets without disrupting homeostatic or regenerative populations is technically demanding. Furthermore, adult tissue architecture, immune maturity, and sustained mechanical loading create a microenvironment fundamentally distinct from that of fetal skin. Thus, effective reprogramming will likely require combinatorial strategies integrating lineage modulation [27,131], transient mechanotransductive recalibration, ECM remodeling, and epigenetic priming.

Collectively, current therapeutic attempts suggest that adult fibroblasts retain latent regenerative potential. Rather than fully recreating the fetal milieu, successful interventions may depend on disrupting the self-reinforcing profibrotic circuit linking Engrailed-1-dependent lineage identity, TGF-β amplification, cytoskeletal tension, and matrix stiffening. By weakening this circuitry in a controlled manner, regenerative healing in adult human skin may become a clinically achievable goal. 

## Figures and Tables

**Table 1:** The most well-established differences between ENF and EPF properties.

Properties	ENFs (En1 Lineage-Negative Fibroblasts)	EPFs (En1 + Lineage-Positive Fibroblasts)
Developmental Timing	Early embryonic dermis; predominant before mid-gestation [19,20]	Emerge later in development; dominant postnatally [19,20]
Functional Role in Wounds	Regenerative; support restoration of dermal architecture and appendages [19,21,107]	Profibrotic; major contributors to scar ECM and myofibroblast formation [19,107,108,109]
Transcriptional Signatures	Developmental genes, ECM remodeling, and reduced contractile gene expression [19]	High collagen I expression, crosslinking enzymes, contractile machinery, myofibroblast markers [19]
Mechanotransduction Sensitivity	Lower YAP/TAZ activation; tolerant of soft/low-tension environments [107]	High YAP/TAZ activation; responsive to stiffness and tension; mechanosensitive profibrotic signaling [107]
Typical ECM Deposition	Produce loose, fetal-like matrix rich in HA and collagen III [17]	Produce dense, aligned, collagen-I-rich ECM with greater crosslinking [19]
Spatial Localization (Adult)	Reduced pool; more papillary-like transcriptional identity [31]	Expanded pool; more reticular-like/deep dermal identity [31]
Plasticity/Reprogrammability	High plasticity, fetal-like	Relatively fixed, stabilized by epigenetic and mechanical cues [107]
Response to Injury	Promote remodeling and reconstitution [19,20,92]	Drive fibrosis, contraction, and scar deposition [19,20,92]

## Data Availability

No new data were created or analyzed in this study. Data sharing is not applicable.

## Abbreviations

The following abbreviations are used in this manuscript:

ECM	extracellular matrix
EPFs	Engrailed-1-positive fibroblasts
TGF-β	transforming growth factor-β
HA	hyaluronic acid
FN	fibronectin
PDGF	platelet-derived growth factor
TNFα	tumor necrosis factor α
IL	interleukin
VEGF	vascular endothelial growth factor
PRP	platelet-rich plasma
MHC	major histocompatibility complex
GAGs	glycosaminoglycans
CS	chondroitin sulfate
MMPs	matrix metalloproteinases
LH	lysyl hydroxylase
PH	prolyl hydroxylase
LOX	low lysyl oxidase
TGM2	transglutaminase 2
FGFs	fibroblast growth factors
FAK	focal adhesion kinase
PI3K	Phosphatidylinositol 3-Kinase
MAPK	Mitogen-Activated Protein Kinase
YAP	yes-associated protein
TAZ	transcriptional coactivator with PDZ-binding motif
CTGF	connective tissue growth factor
SOX	SRY-related high-mobility-group (HMG) box
Trps1	Trichorhinophalangeal Syndrome Type I
PDGFRA	PDGF receptor A
DLK-1	delta-like homology-1
LRIG1	leucine-rich repeat protein
Twist-2/Dermo-1	twist-related protein-2
Prrx1	pair-related homeobox 1
PPFs	pair-related homeobox 1 (Prrx1)-positive fibroblasts
PNFs	pair-related homeobox 1 (Prrx1)-negative fibroblasts
